# Trajectories of Glycemia Following Acute Pancreatitis: A Prospective Longitudinal Study

**DOI:** 10.7759/cureus.117035

**Published:** 2026-09-27

**Authors:** Uppati V Krishna, Bommi Gowrishankar, Mutyala Trivenu V Chowdary, Vamsi D Yadav, LRS Girinadh, Ravada Kinneresh

**Affiliations:** 1 Department of Gastroenterology, Great Eastern Medical School and Hospital, Srikakulam, IND; 2 Department of General Medicine, Great Eastern Medical School and Hospital, Srikakulam, IND; 3 Department of Gastroenterology, Andhra Medical College, Visakhapatnam, IND; 4 Department of Dentistry, Great Eastern Medical School and Hospital, Srikakulam, IND

**Keywords:** acute pancreatitis, diabetes mellitus, glycated hemoglobin, longitudinal study, prediabetes, type 3c

## Abstract

Introduction: Acute pancreatitis (AP) may cause persistent disturbances in glucose metabolism that extend beyond the resolution of acute inflammatory episodes. However, the progression of glycemic abnormalities during the early recovery period remains insufficiently characterized, emphasizing the need for prospective longitudinal evaluation. We aimed to evaluate glycemia trajectories after an episode of AP over a 12-month follow-up by determining the cumulative incidence of new-onset prediabetes and diabetes and assessing longitudinal changes in glycemic parameters.

Methods: This prospective longitudinal observational study included 77 adults diagnosed with AP without preexisting diabetes who were followed up for 12 months. We recorded clinical characteristics, pancreatitis severity scores, laboratory investigations, and fasting blood glucose (FBG), postprandial blood glucose (PPBG), and glycated hemoglobin (HbA1c) levels at baseline, six months, and 12 months. Statistical analyses were performed using repeated-measures analysis of variance for longitudinal comparisons, Bonferroni-adjusted post hoc tests, and McNemar's test for categorical outcomes. Statistical significance was set at p < 0.05.

Results: Among the 77 participants, 45 (58.4%) were male participants, and 39 (50.6%) had biliary pancreatitis. During follow-up, the incidence of prediabetes increased significantly from 12 (15.6%) at six months to 19 (24.7%) at 12 months (p = 0.023), while that of diabetes increased from three (3.9%) to five (6.5%); however, the difference was not statistically significant (p = 0.480). Overall, glycemic abnormalities increased significantly from 15 (19.5%) to 24 (31.2%) (p = 0.008). Repeated-measures analysis demonstrated a significant increase in FBG levels from 96 ± 9.5 to 106 ± 17.0 mg/dL (p < 0.001), PPBG from 127 ± 16.5 to 150 ± 35.0 mg/dL (p = 0.003), and HbA1c from 5.52% ± 0.11% to 6.06 ± 0.40% (p < 0.001) over the 12-month period. Glycemic trajectory analysis showed that 35 (45.5%) participants remained in the normal-stable group, 35 (45.5%) exhibited a moderately stable pattern, and seven (9.1%) demonstrated a high glycemic trajectory.

Conclusion: Progressive deterioration in glycemic control occurs during the first year following AP, with nearly one-third of the patients developing prediabetes or diabetes. HbA1c demonstrated the greatest longitudinal increase and may serve as a sensitive marker for identifying patients at risk for post-pancreatitis dysglycemia. These findings support routine metabolic surveillance after AP to facilitate early diagnosis and timely intervention.

## Introduction

Acute pancreatitis (AP) is one of the most common gastrointestinal emergencies and is characterized by acute inflammation of the pancreas with varying degrees of local tissue injury and a systemic inflammatory response [1]. Although most patients recover from an acute episode, increasing evidence suggests that AP is not merely a transient inflammatory disorder but may have significant long-term metabolic consequences, particularly involving glucose homeostasis [2].

Diabetes mellitus secondary to diseases of the exocrine pancreas, commonly referred to as post-pancreatitis diabetes mellitus (PPDM) or type 3c diabetes mellitus, has emerged as an important clinical entity [3]. Approximately 80% of PPDM cases of pancreatogenic diabetes are attributed to pancreatitis [4]. Among adults with newly diagnosed diabetes, diabetes secondary to diseases of the exocrine pancreas or pancreatogenic diabetes represents 1.6%-1.8% of cases, compared with 1.1% for type 1 diabetes mellitus [5]. Unlike type 2 diabetes mellitus, PPDM is associated with more severe disturbances in glycemic control, higher glycated hemoglobin (HbA1c) levels, increased insulin deficiency, and poorer long-term outcomes, including greater all-cause mortality. These differences emphasize the need for early recognition and appropriate follow-up of AP [6].

The development of dysglycemia following AP is multifactorial in nature. Pancreatic inflammation may result in the destruction of insulin-producing β-cells, disruption of glucagon secretion, persistent systemic inflammation, insulin resistance, impaired incretin response, and stress-induced hyperglycemia [7]. Although pancreatic necrosis contributes to endocrine dysfunction, recent evidence indicates that new-onset diabetes may occur even in patients with mild AP, suggesting that factors other than structural pancreatic damage influence disease progression [8].

Several systematic reviews have demonstrated that approximately one-third of patients develop prediabetes or diabetes after an episode of AP, with the risk increasing progressively during follow-up [9,10]. However, most available studies are retrospective or cross-sectional, and longitudinal data evaluating glycemic trajectories at predefined intervals remain limited. Consequently, current clinical guidelines do not clearly define optimal strategies for glucose surveillance following AP, leading to delayed diagnosis and missed opportunities for early intervention [9].

This study aimed to evaluate the development and progression of post-pancreatitis dysglycemia during the first 12 months after an episode of AP. The primary objective was to determine the cumulative incidence of new-onset prediabetes and diabetes at 6 and 12 months following AP. The secondary objectives were to assess longitudinal changes in fasting blood glucose (FBG), postprandial blood glucose (PPBG), and HbA1c from baseline to 6 and 12 months, and to characterize longitudinal glycemic trajectory patterns based on changes in these glycemic parameters during the 12-month follow-up period.

## Materials and methods

This prospective, observational, longitudinal study was conducted in the Department of Gastroenterology, Andhra Medical College, Visakhapatnam, India, over 16 months, from July 2023 to October 2024. Adult patients diagnosed with AP were consecutively recruited after obtaining their written informed consent. The diagnosis of AP was established according to standard clinical criteria requiring at least two of the following three features: characteristic abdominal pain suggestive of AP; serum amylase and/or lipase levels at least three times the upper limit of normal; and radiological evidence of AP on computed tomography, magnetic resonance imaging, or ultrasonography [11]. Participants were prospectively followed from hospital admission through 12 months after discharge, with clinical evaluations scheduled at baseline, six months, and 12 months.

The study included adults aged 18 years or older who were diagnosed with AP and were willing to participate. Patients with preexisting diabetes mellitus, chronic pancreatitis, disseminated malignancy, pregnancy, liver cirrhosis, chronic kidney disease, or other chronic illnesses known to influence glucose metabolism were excluded. We also excluded individuals younger than 18 years or older than 80 years and those unwilling to provide informed consent.

The sample size was estimated using G*Power software (version 3.1.9.7; Heinrich-Heine-Universität Düsseldorf, Düsseldorf, Germany), based on the findings of a previous prospective longitudinal study that reported a 43% incidence of glycemic abnormalities following AP [12]. Assuming a statistical power of 80% (β = 0.20), a two-sided alpha error of 5% (α = 0.05), and a margin of error of 5%, the minimum required sample size was calculated as 70 participants. To compensate for potentially incomplete data, participant attrition, and loss to follow-up during the 12-month observation period, the sample size was increased by 10%, resulting in a final target sample of 77 participants.

Demographic characteristics, including age, sex, body mass index (BMI), waist circumference, and hip circumference, were recorded at baseline. Clinical information, including pancreatitis severity assessed using the Bedside Index for Severity in Acute Pancreatitis (BISAP) [13] and Acute Physiology and Chronic Health Evaluation II (APACHE II) scores [14], presence of pancreatic necrosis, recurrence of pancreatitis, and underlying etiology were documented from hospital records. Laboratory investigations included HbA1c, FBG, PPBG, homeostatic model assessment for insulin resistance (HOMA-IR) [15], lipid profile, liver function tests, complete blood count, C-reactive protein, and serum ferritin levels. Glycemic parameters were assessed at baseline, six months, and 12 months. FBG was measured after overnight fasting, PPBG was measured two hours after a standardized meal, and HbA1c was measured by high-performance liquid chromatography using a D-10 hemoglobin testing system (Bio-Rad Laboratories, Hercules, CA).

Diabetes was diagnosed according to the established diagnostic criteria based on fasting plasma glucose, two-hour postprandial/oral glucose tolerance test values, and/or HbA1c levels [16]. Normoglycemia was defined as HbA1c <5.7% and FBG level <100 mg/dL. New-onset prediabetes was defined as HbA1c between 5.7% and 6.4% and/or FBG between 100 and 125 mg/dL at either the 6- or 12-month follow-ups. New-onset diabetes was defined as HbA1c ≥6.5% and/or FBG ≥126 mg/dL during follow-up [17]. In addition to these binary diagnostic categories, participants were classified into three mutually exclusive glycemic trajectory groups that characterized the temporal pattern of glucose metabolism over the 12-month follow-up period using the same conceptual framework described by Bharmal et al. [12].

Longitudinal values of HbA1c (primary marker), FBG, and PPBG measured at baseline, six months, and 12 months were examined. Participants were assigned to the normal-stable trajectory if HbA1c remained <5.7% (and FBG remained <100 mg/dL) at all three time points with no clinically meaningful upward trend; to the moderate-stable trajectory if HbA1c remained predominantly in the 5.7%-6.4% range (or FBG 100-125 mg/dL) without a progressive rise into the diabetic range; and to the high-increasing trajectory if there was a clear progressive rise culminating in HbA1c ≥6.5% and/or FBG ≥126 mg/dL by 12 months.

Assignment was performed independently by two investigators, and discrepancies were resolved by consensus. The resulting groups comprised 35 (45.5%) normal-stable, 35 (45.5%) moderate-stable, and seven (9.1%) high-increasing participants. The primary outcome of the study was the cumulative incidence of new-onset prediabetes and diabetes at 6 and 12 months after an episode of AP. Secondary outcomes included longitudinal changes in FBG, PPBG, and HbA1c levels, as well as characterization of glycemic trajectories throughout the follow-up period.

The study protocol was approved by the Institutional Ethics Committee before participant recruitment (138/IEC AMC/JUN 2023, June 15, 2023). Written informed consent was obtained from all participants before enrolment. Participation was voluntary, confidentiality of patient information was maintained throughout the study, and participants were free to withdraw from the study at any stage, without affecting their clinical care. The study imposed no financial burden on participants and received no external funding.

Participants were prospectively followed for 12 months after discharge, with planned assessments at baseline, six months, and 12 months. Follow-up visits were scheduled at the predefined assessment time points, and clinical and laboratory data were recorded at each assessment. All 77 enrolled participants completed the 12-month follow-up and were included in the final analysis; therefore, there were no losses to follow-up. Glycemic assessments were performed at each scheduled visit, and no imputation was required for missing laboratory measurements in the final analytical dataset. Clinical and pancreatitis-related variables, including BMI, pancreatitis severity scores, pancreatic necrosis, recurrence, and etiology, were collected as baseline clinical characteristics; however, they were not included in predictive modeling or formal subgroup analyses, as the primary objective was to evaluate the longitudinal development of post-pancreatitis dysglycemia and glycemic trajectory patterns over the 12-month follow-up period.

We performed statistical analyses using IBM Statistical Package for the Social Sciences Statistics for Windows (version 25.0; IBM Corp., Armonk, NY). The normality of continuous variables was assessed using the Shapiro-Wilk test. Continuous variables demonstrating a normal distribution were expressed as mean ± standard deviation, whereas categorical variables were summarized as frequencies and percentages. Longitudinal changes in FBG, PPBG, and HbA1c levels at baseline, six months, and 12 months were analyzed using repeated-measures analysis of variance (ANOVA). Mauchly's test of sphericity was performed, and the Greenhouse-Geisser correction was applied when the assumption of sphericity was violated. Pairwise post hoc comparisons were conducted using Bonferroni-adjusted paired t-tests. We analyzed changes in the incidence of new-onset prediabetes and diabetes between the 6- and 12-month follow-up visits using McNemar's test with Yates’ continuity correction. All statistical tests were two-tailed, and statistical significance was set at p < 0.05.

## Results

We assessed 91 patients with AP for eligibility between July 2023 and October 2024. After excluding 14 (15.4%) participants, 77 (84.6%) patients with AP without diabetes were enrolled and underwent baseline clinical examination, pancreatitis severity assessment, and glycemic profiling. At Baseline, 22 (28.6%) patients with HbA1c of 5.7%-6.4% and 55 (71.4%) patients with HbA1c <5.7% were identified. New-onset diabetes and prediabetes were documented during follow-up. After further exclusion, five (6.5%) patients developed new-onset diabetes, and 19 (24.7%) developed new-onset prediabetes at 12 months, yielding 24 (31.2%) patients with new-onset dysglycemia (Figure 1).

**Figure 1:**
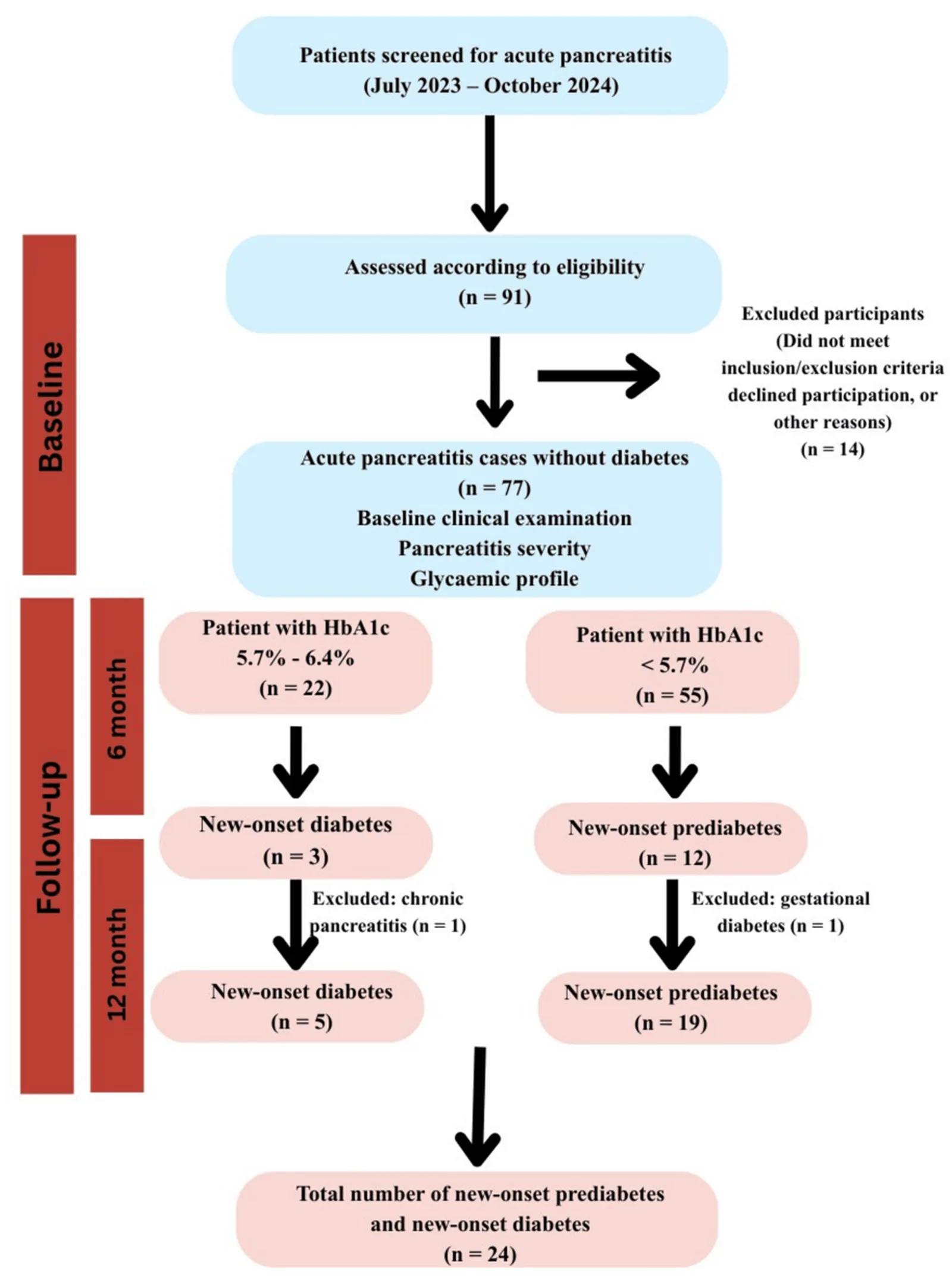
Study flowchart HbA1c: glycated hemoglobin Image credit: The image was created using Canva (Canva Pty Ltd., Sydney, Australia)

The study cohort had a mean BMI of 27.20 ± 3.87 kg/m², with mean waist and hip circumferences of 95.90 ± 7.60 and 97.70 ± 9.09 cm, respectively. This study included 45 men (58.40%) and 32 women (41.60%). The mean BISAP score was 0.9 ± 0.8, and the mean APACHE II score was 6.00 ± 1.60, indicating a predominantly mild disease severity. Pancreatic necrosis was observed in four (5.19%) participants, whereas 22 (28.60%) experienced recurrent pancreatitis during follow-up. Biliary pancreatitis was the most common etiology, followed by alcohol-related pancreatitis. Baseline laboratory investigations demonstrated a mean HbA1c of 5.32% ± 0.38%, HOMA-IR of 1.40 ± 0.50, total cholesterol of 154.00 ± 20.50 mg/dL, and C-reactive protein level of 9.00 ± 20.75 mg/L (Table 1).

**Table 1:** Demographic and clinical characteristics of the study participants (n = 77) Data are presented as mean ± SD for continuous variables and frequency (percentage) for categorical variables Reference ranges used by the study laboratory for adult population are provided BMI: body mass index, BISAP: Bedside Index for Severity in Acute Pancreatitis, APACHE II: Acute Physiology and Chronic Health Evaluation II, HbA1c: glycated hemoglobin, HOMA-IR: homeostatic model assessment of insulin resistance, HDL: high-density lipoprotein, LDL: low-density lipoprotein, GGT: gamma-glutamyl transferase, ALP: alkaline phosphatase, AST: aspartate aminotransferase, ALT: alanine aminotransferase, CRP: C-reactive protein, SD: standard deviation

Parameter		Values	Normal reference range
Anthropometrics, mean ± SD	BMI (kg/m^2^)	27.20 ± 3.87	18.5-24.9
	Waist circumference (cm)	95.90 ± 7.60	<94 cm (men); <80 cm (women)
	Hip circumference (cm)	97.70 ± 9.09	-
Sex, n (%)	Male	45 (58.40)	-
	Female	32 (41.60)	-
Pancreatitis-related, mean ± SD	BISAP score	0.9 ± 0.8	0-5 (lower score indicate severity)
	APACHE II score	6.00 ± 1.60	0-71 (lower score indicate severity)
Pancreatic necrosis, n (%)	Yes	4 (5.19)	-
	No	73 (94.81)	-
Pancreatitis recurrence, n (%)	Yes	22 (28.60)	-
	No	55 (71.40)	-
Pancreatitis etiology, n (%)	Biliary	39 (50.60)	-
	Alcohol-related	18 (23.40)	-
Laboratory findings, mean ± SD	HbA1c (mmol/mol)	5.32 ± 0.38	20-41 mmol/mol (nondiabetic range)
	HOMA-IR	1.40 ± 0.50	<2.5
Lipid profile, mean ± SD	Total cholesterol (mg/dL)	154.00 ± 20.50	<200 mg/dL
	HDL (mg/dL)	73.48 ± 22.80	≥40 mg/dL (men); ≥50 mg/dL (women)
	LDL (mg/dL)	88.00 ± 27.30	<100 mg/dL
	Triglycerides (mg/dL)	101.80 ± 33.00	<150 mg/dL
Liver panel, mean ± SD	Bilirubin (µmol/L)	16.00 ± 10.30	5-21 µmol/L
	GGT (U/L)	85.00 ± 146.00	8-61 U/L (men); 5-36 U/L (women)
	ALP (U/L)	90.00 ± 29.00	44-147 U/L
	AST (U/L)	41.00 ± 63.50	10-40 U/L
	ALT (U/L)	52.00 ± 57.75	7-56 U/L (men); 7-35 U/L (women)
Markers of inflammation, mean ± SD	White blood cell count (10^9^/L)	9.10 ± 2.65	4.0-11.0 × 10⁹/L
	Basophils (10^9^/L)	0.20 ± 0.10	0.0-0.1 × 10⁹/L
	Eosinophils (10^9^/L)	0.10 ± 0.10	0.0-0.5 × 10⁹/L
	Neutrophils (10^9^/L)	6.90 ± 2.95	2.0-7.5 × 10⁹/L
	Monocytes (10^9^/L)	0.80 ± 0.25	0.2-0.8 × 10⁹/L
	Lymphocytes (10^9^/L)	1.70 ± 0.55	1.0-4.0 × 10⁹/L
	CRP (mg/L)	9.00 ± 20.75	<5 mg/L
	Ferritin (ng/mL)	252.50 ± 146.15	24-336 ng/mL (men); 11-307 ng/mL (women)

The cumulative incidence of glycemic abnormalities increased during the follow-up period. At six months, 12 participants (15.58%) developed prediabetes, which increased to 19 (24.68%) at 12 months, representing a statistically significant increase (χ² = 5.14, p = 0.023). New-onset diabetes increased from three (3.90%) participants at six months to five (6.49%) participants at 12 months; however, this increase was not statistically significant (χ² = 0.50, p = 0.480). Overall, the proportion of participants with any glycemic abnormality increased significantly from 19.48% at six months to 31.17% at 12 months (χ² = 7.11, p = 0.008) (Table 2).

**Table 2:** Incidence of new-onset prediabetes and diabetes during the 12-month follow-up Data are presented as frequencies (percentages). Comparisons between the 6- and 12-month follow-ups were performed using McNemar's test with Yates continuity correction ^*^p < 0.05 was considered statistically significant

New onset	6 months (n = 77)	12 months (n = 77)	Test value (χ²)	p value
Prediabetes, n (%)	12 (15.68%)	19 (24.67%)	5.14	0.023^*^
Diabetes, n (%)	3 (3.89%)	5 (6.49%)	0.50	0.480
Total, n (%)	15 (19.48%)	24 (31.16%)	7.11	0.008^*^

Repeated-measures ANOVA demonstrated a significant longitudinal deterioration in the glycemic parameters during the 12-month follow-up period. Mean FBG increased significantly from 96 ± 9.5 mg/dL at baseline to 106 ± 12.5 mg/dL at six months and remained at 106 ± 17.0 mg/dL at 12 months (F = 7.81, p < 0.001). PPBG also showed a significant progressive increase from 127 ± 16.5 mg/dL at baseline to 137 ± 14.5 mg/dL at six months and 150 ± 35.0 mg/dL at 12 months (F = 6.43, p = 0.003). The greatest change was observed for HbA1c, which increased significantly from 5.52 ± 0.11% at baseline to 5.72 ± 0.15% at six months and 6.06 ± 0.40% at 12 months (F = 31.89, p < 0.001) (Table 3).

**Table 3:** Longitudinal comparison of glycemic parameters at baseline, six months, and 12 months among participants with new-onset prediabetes or diabetes (n = 24) using repeated-measures analysis of variance Data are presented as mean ± SD with 95% CI. Longitudinal comparisons were performed using repeated measures ANOVA Analysis was restricted to the subgroup of participants who developed new-onset prediabetes or diabetes during follow-up (n = 24) *p < 0.05 was considered statistically significant SD: standard deviation, CIs: confidence intervals, ANOVA: analysis of variance, HbA1c: glycated hemoglobin

Parameters	Timeline	Mean ± SD	95% CI		Test value	p value
			Lower	Upper		
Fasting blood glucose (mg/dL), n = 24	Baseline	96.0 ± 9.5	92.0	100.0	7.81	<0.001^*^
	6 months	106.0 ± 12.5	100.7	111.3		
	12 months	106.0 ± 17.0	98.8	113.2		
Postprandial blood glucose (mg/dL), n = 24	Baseline	127.0 ± 16.5	120.0	134.0	6.43	0.003^*^
	6 months	137.0 ± 14.5	130.9	143.1		
	12 months	150.0 ± 35.0	135.2	164.8		
HbA1c (%), n = 24	Baseline	5.52 ± 0.11	5.47	5.57	31.89	<0.001^*^
	6 months	5.72 ± 0.15	5.66	5.78		
	12 months	6.06 ± 0.40	5.89	6.23		

Bonferroni-adjusted post hoc comparisons revealed that FBG increased significantly from baseline to both six months (p = 0.012) and 12 months (p = 0.015), whereas no significant difference was observed between the 6- and 12-month assessments (p = 1.000). PPBG levels demonstrated a significant increase only between baseline and 12 months (p = 0.004), whereas comparisons between baseline and six months (p = 0.028) and between 6 and 12 months (p = 0.086) were not statistically significant after Bonferroni correction. HbA1c levels significantly increased across all follow-up intervals, including baseline to six months (p = 0.003), baseline to 12 months (p < 0.001), and 6-12 months (p < 0.001), indicating the progressive worsening of long-term glycemic control throughout the study period (Table 4).

**Table 4:** Pairwise comparisons of glycemic parameters between follow-up time points among participants with new-onset prediabetes or diabetes (n = 24) with Bonferroni correction Pairwise comparisons were performed using paired t-tests with Bonferroni correction following repeated measures analysis of variance Analysis restricted to the subgroup of participants who developed new-onset prediabetes or diabetes during follow-up (n = 24) ^*^Statistical significance was set at p < 0.017 after Bonferroni adjustment HbA1c: glycated hemoglobin

Parameter	Comparison	Mean difference	t value	p value (adjusted)
Fasting blood glucose	Baseline vs. 6 months	10.00	31.2	0.012^*^
	Baseline vs. 12 months	10.00	2.79	0.015^*^
	6 months vs. 12 months	0.00	0.00	1.000
Postprandial blood glucose	Baseline vs. 6 months	10.00	2.38	0.028
	Baseline vs. 12 months	23.00	3.39	0.004^*^
	6 months vs. 12 months	13.00	1.93	0.086
HbA1c	Baseline vs. 6 months	0.20	3.33	0.003^*^
	Baseline vs. 12 months	0.54	6.75	<0.001^*^
	6 months vs. 12 months	0.34	4.86	<0.001^*^

Analysis of glycemic trajectories demonstrated that 35 (45.45%) participants maintained a normal glycemic profile, 35 (45.45%) participants exhibited a moderately stable glycemic pattern, and seven (9.10%) participants showed a high glycemic trajectory over the 12-month follow-up. Overall, FBG, PPBG, and HbA1c levels demonstrated progressive increases, with HbA1c showing the most pronounced elevation, reflecting cumulative deterioration in glycemic control following AP (Figure 2).

**Figure 2:**
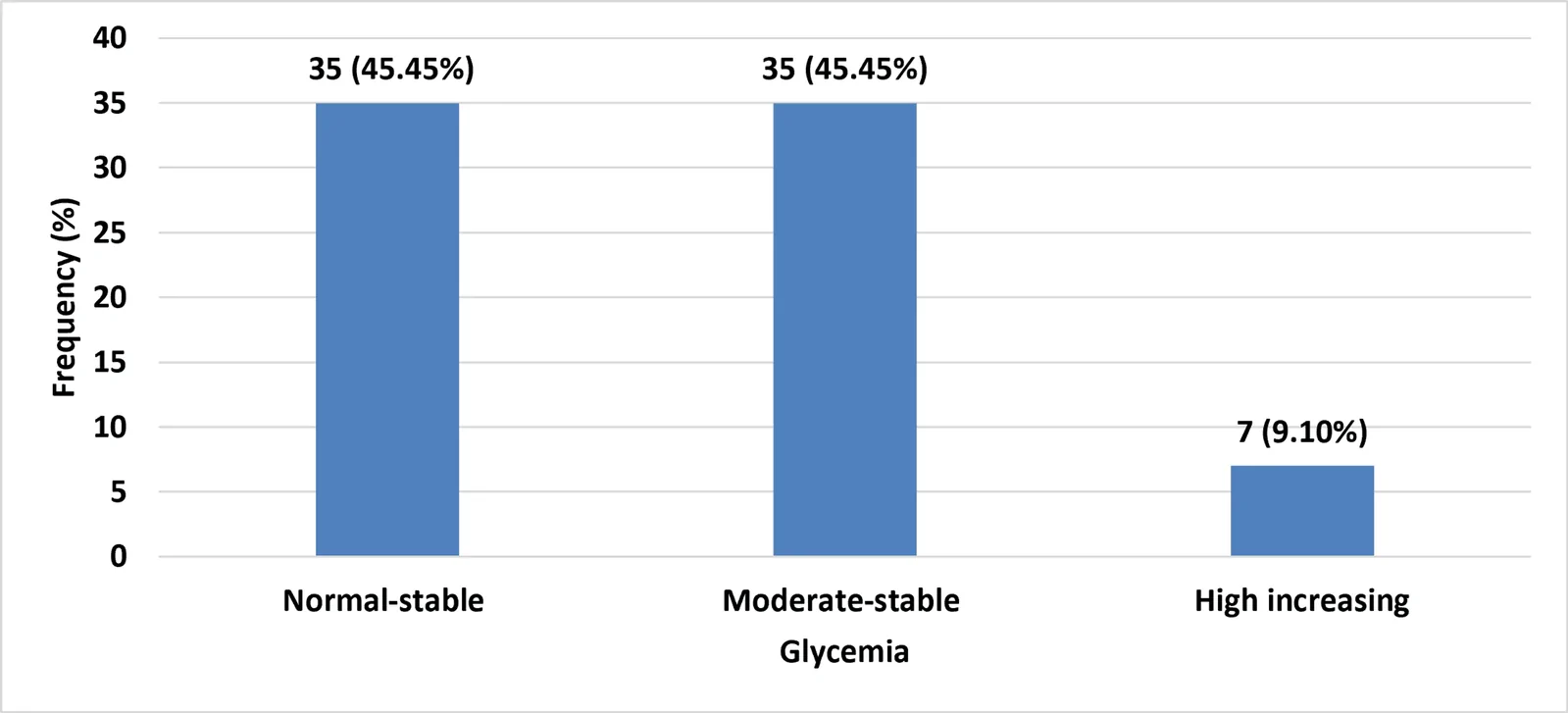
Glycemic status of study population over 12-month period

## Discussion

This prospective longitudinal study evaluated the trajectory of glycemia during the first year after an episode of AP and showed progressive deterioration in glucose metabolism despite recovery from acute illness. Nearly one-third of the study population developed either prediabetes or diabetes within 12 months, accompanied by significant increases in FBG, PPBG, and HbA1c levels. These findings reinforce the concept that AP is not merely an acute inflammatory event but may initiate long-term endocrine dysfunction that predisposes patients to PPDM [2].

The baseline characteristics of the study population reflected the epidemiological profiles commonly reported for AP. The majority of the patients were men, biliary pancreatitis was the predominant etiology, and only a small proportion had pancreatic necrosis, suggesting that most participants had mild to moderately severe disease. Nevertheless, more than one-fourth of the patients experienced recurrent pancreatitis during follow-up. These observations are clinically important because recurrent pancreatic inflammation is increasingly being recognized as an independent determinant of progressive endocrine insufficiency [18]. A recent meta-analysis by Zahariev et al. [19] demonstrated that recurrent AP, pancreatic necrosis, obesity, and alcohol-related pancreatitis significantly increase the risk of developing PPDM. Although pancreatic necrosis was infrequent in our cohort, recurrent inflammatory episodes may have contributed substantially to the progressive deterioration in glycemic control observed during follow-up.

One of the most important findings of the present study was the significant increase in the cumulative incidence of prediabetes from 15.6% at six months to 24.7% at 12 months, whereas diabetes increased from 3.9% to 6.5%; however, the difference was not statistically significant. Overall, almost one-third of the patients developed abnormal glucose metabolism within one year of AP. These findings are consistent with those of a prospective cohort study by Bharmal et al. [12], who demonstrated that glycemic abnormalities progressively increase during the first two years following AP, with many patients initially developing prediabetes before progressing to overt diabetes. Similarly, a systematic review by Das et al. [9] and a more recent meta-analysis by Zahariev et al. [19] reported that approximately 30%-40% of patients eventually develop prediabetes or diabetes after an episode of AP. The slightly lower incidence observed in the present study may be attributable to the shorter follow-up duration of 12 months, lower prevalence of pancreatic necrosis, and predominance of mild disease compared with previous cohorts. Conversely, earlier studies reported a substantially higher incidence of diabetes, particularly among patients with severe necrotizing pancreatitis [20,21]. These discrepancies are likely explained by differences in disease severity, follow-up duration, patient demographics, and the diagnostic criteria used across studies.

This study demonstrated a significant longitudinal increase in FBG, PPBG, and HbA1c levels. Interestingly, FBG increased significantly during the first six months and subsequently reached a plateau, whereas PPBG exhibited a continuous increase throughout the follow-up period. HbA1c showed the greatest and most consistent increase across all time points, indicating progressive deterioration of long-term glycemic control. These findings suggest that disturbances in postprandial glucose metabolism and chronic glycemic exposure develop gradually, even after apparent clinical recovery from AP [22]. Similar temporal patterns were reported by Bharmal et al. [12], who identified distinct glycemic trajectories following AP and showed that HbA1c was the most reliable marker for identifying individuals at risk of persistent dysglycemia.

Several pathophysiological mechanisms might explain this observation. Acute pancreatic inflammation results in varying degrees of destruction of insulin-producing β-cells, thereby reducing insulin secretory capacity [22]. Simultaneously, persistent systemic inflammation induces insulin resistance through cytokine-mediated impairment of the insulin signaling pathways [7]. Furthermore, disruption of the incretin axis, involving glucagon-like peptide-1 and glucose-dependent insulinotropic peptide, contributes to impaired postprandial insulin secretion, which explains the pronounced increase in postprandial glucose observed in the present study [7,22]. These mechanisms are supported by a review by Walker et al. [23], who emphasized that post-pancreatitis diabetes results from a combination of endocrine insufficiency, exocrine dysfunction, chronic inflammation, and insulin resistance, rather than isolated β-cell destruction. Similarly, Yang et al. [24] demonstrated that stress hyperglycemia during AP predicts persistent metabolic dysfunction and adverse clinical outcomes, suggesting that early glucose abnormalities may identify individuals at increased risk of long-term diabetes.

Another notable finding was that HbA1c levels increased significantly between each follow-up interval, whereas fasting and postprandial glucose levels exhibited less consistent changes after adjustment for multiple comparisons. HbA1c reflects the average glycemic exposure over approximately three months and is less susceptible to short-term biological fluctuations than fasting or postprandial glucose measurements. Therefore, the consistent increase in HbA1c suggests that progressive impairment of glucose homeostasis continues even after the resolution of the acute inflammatory phase. This observation supports current recommendations advocating serial HbA1c monitoring in patients recovering from AP to facilitate early detection of PPDM [25]. The glycemic trajectory analysis further demonstrated that although the majority of patients remained within the normal-stable or moderate-stable trajectory groups, approximately one-tenth exhibited a high glycemic trajectory. Identification of this subgroup has important clinical implications because these individuals are likely to benefit from intensive metabolic surveillance and early preventive interventions before irreversible endocrine dysfunction develops [26].

Previous studies have identified several clinical and biochemical features during hospitalization that may help identify patients at increased risk of subsequent metabolic dysfunction after AP. In particular, pancreatic necrosis and greater disease severity have been associated with an increased risk of post-pancreatitis diabetes, while stress hyperglycemia during AP has also been associated with persistent metabolic dysfunction and adverse clinical outcomes [17-19,22]. These findings suggest that early clinical and glycemic characteristics during the index hospitalization may help identify patients who require closer metabolic surveillance after discharge. Incorporating these characteristics with post-discharge HbA1c and fasting glucose monitoring may facilitate more individualized metabolic surveillance. However, these factors were not specifically evaluated as independent predictors in the present study and should therefore be interpreted in the context of evidence from previous cohorts.

Clinical implications

The findings emphasize that patients recovering from AP require structured long-term metabolic follow-up, regardless of initial disease severity. Routine assessment of FBG and HbA1c at regular intervals, particularly during the first year after AP, may facilitate early identification of post-pancreatitis dysglycemia. Patients with recurrent pancreatitis, alcohol-related disease, obesity, or pancreatic necrosis should be considered at higher risk and may benefit from closer surveillance, lifestyle modifications, nutritional counseling, and timely endocrinology referral. Previous studies have additionally identified marked in-hospital glycemic variability, hyperglycemia at admission, severe pancreatic necrosis, and peak in-hospital glucose levels >200 mg/dL as clinical features associated with an increased risk of subsequent metabolic dysfunction. These parameters may therefore help identify patients who require more intensive metabolic surveillance after discharge. Early recognition and management of prediabetes may delay or prevent progression to overt PPDM.

Limitations

This study had several limitations. First, it was conducted at a single tertiary care center with a relatively modest sample size, which may limit the generalizability of the findings. Second, the follow-up period was limited to 12 months and may therefore have underestimated the long-term incidence of PPDM, which may continue to increase over subsequent years. Third, pancreatic endocrine function was evaluated primarily using conventional glycemic markers, without assessment of C-peptide, insulin secretory capacity, or incretin hormones. Finally, subgroup analyses according to disease severity, pancreatic necrosis, and etiology were not adequately powered because of the relatively small number of affected participants. In addition, we classified glycemic trajectory patterns based on longitudinal changes in glycemic parameters, which may have involved some degree of subjectivity.

Future research

Future research should focus on improving early identification of patients at risk of post-pancreatitis dysglycemia by evaluating circulating biomarkers and integrated risk-stratification approaches. Potential candidates include specific extracellular vesicles, bile acid profiles and related metabolic mediators such as fibroblast growth factor 19, as well as genetic risk scores. Prospective studies incorporating these biomarkers together with clinical characteristics and early in-hospital glycemic patterns may help establish more accurate approaches for identifying individuals at risk before persistent metabolic abnormalities develop. In addition, randomized controlled trials are needed to determine whether targeted early post-discharge metabolic interventions can prevent or delay progression to prediabetes and diabetes. Particular areas of interest include early exocrine pancreatic enzyme replacement therapy and selected incretin-based therapies. Such trials should evaluate appropriate patient selection, timing of intervention, metabolic outcomes, safety, and long-term progression to post-pancreatitis diabetes.

## Conclusions

In conclusion, glycemic abnormalities increased progressively during the first year following AP, with a gradual increase in the proportion of patients developing prediabetes and diabetes and worsening glycemic parameters over follow-up. These findings suggest progressive deterioration in glycemic control and glucose metabolism following AP. However, we did not assess direct measures of pancreatic endocrine function; therefore, the observed changes should not be interpreted as definitive evidence of progressive deterioration in pancreatic endocrine function. Longer term studies incorporating direct measures of β-cell function are warranted to further elucidate the mechanisms underlying post-pancreatitis dysglycemia.
